# Withdrawal of inhaled corticosteroids can be safe in COPD patients at low risk of exacerbation: a real-life study on the appropriateness of treatment in moderate COPD patients (OPTIMO)

**DOI:** 10.1186/1465-9921-15-77

**Published:** 2014-07-08

**Authors:** Andrea Rossi, Massimo Guerriero, Antonio Corrado

**Affiliations:** 1 Pulmonary Unit, General Hospital and University of Verona, Verona, Italy; 2 Department of Computer Science, University of Verona, Verona, Italy; 3 Respiratory Intensive Care Unit and Thoracic Physiopathology, University and General Hospital Careggi, Florence, Italy

**Keywords:** COPD, Inhaled corticosteroids, Bronchodilators, Exacerbations

## Abstract

**Background:**

It has been suggested that withdrawal of inhaled corticosteroids (ICS) in COPD patients on maintenance treatment results in deterioration of symptoms, lung function and exacerbations. The aim of this real-life, prospective, multicentric study was to investigate whether withdrawal of ICS in COPD patients at low risk of exacerbation is linked to a deterioration in lung function and symptoms and to a higher frequency of exacerbations.

**Methods:**

914 COPD patients, on maintenance therapy with bronchodilators and ICS, FEV_1_>50% predicted, and <2 exacerbations/year were recruited. Upon decision of the primary physicians, 59% of patients continued their ICS treatment whereas in 41% of patients ICS were withdrawn and regular therapy was continued with long-acting bronchodilators mostly (91% of patients). FEV_1_, CAT (COPD Assessment Test), and occurrence of exacerbations were measured at the beginning (T0) and at the end (T6) of the 6 months observational period.

**Results:**

816 patients (89.3%) concluded the study. FEV_1_, CAT and exacerbations history were similar in the two groups (ICS and no ICS) at T0 and at T6. We did not observe any deterioration of lung function symptoms, and exacerbation rate between the two groups at T0 and T6.

**Conclusions:**

We conclude that the withdrawal of ICS, in COPD patients at low risk of exacerbation, can be safe provided that patients are left on maintenance treatment with long-acting bronchodilators.

## Background

Chronic Obstructive Pulmonary Disease (COPD) is a major medical problem [1]. Regular therapy with long-acting bronchodilators (LABA: long-acting beta-adrenergic agonists; LAMA: long-acting antimuscarinic agents) improves lung function, dyspnea and quality of life in symptomatic patients with spirometric evidence of airflow obstruction [2]. In addition, long-acting bronchodilators can reduce the rate of exacerbations due probably to a reduction in pulmonary hyperinflation and a re-setting of lung function dynamics [3]. Additional treatment with inhaled corticosteroids (ICS), in particular the ICS/LABA fixed dose combinations (FDC), has been recommended for symptomatic COPD patients at a high risk of frequent exacerbations, i.e. patients with severe airflow limitation (FEV_1 _<50% predicted) and/or a clinical history of frequent exacerbations (>2/year) [4]. However, several clinical studies have shown that COPD patients with moderate airflow limitation, i.e. FEV_1_ > 50% predicted, and history of less than 2 exacerbations per year, have also being treated with ICS in clinical practice [5-7], although no recommendation for this can be found in either international [2,4,8,9] or national [10] documents. This overtreatment is no trivial matter [11]. In fact, it has been documented that regular treatment with ICS for COPD patients can carry the risk of significant adverse effects [12] particularly regarding an increased risk of pneumonia [13-16]. A simple solution might be to withdraw ICS in patients who do not require it. However, it has been shown that withdrawing ICS in patients on maintenance treatment may increase the risk of exacerbations, not only in comparison to placebo [17-19], but also in comparison to regular treatment with long-acting bronchodilators [20].

In fact, clinicians seem more confident in keeping patients on ICS rather than withdrawing it [21], even though it can be regarded as a form of overtreatment [11] and hence inappropriate according to guidelines. The COSMIC trial [20] recruited patients with severe airflow limitation and frequent exacerbations. Hence, the effects of withdrawal of ICS was not investigated in COPD patients at low risk for exacerbations, such as those with moderate airflow limitation, i.e. FEV_1_>50% predicted and no history of frequent exacerbations, i.e. less than 2 per year. This study was therefore undertaken to assess whether withdrawal of ICS in these COPD patients is safe or whether it is linked to a deterioration in lung function and symptoms and to a higher frequency of exacerbations.

## Methods

Approval was obtained from the ethics committees of each participating centre and written informed consent forms were signed by all the patients.

### Study design

**Figure 1:**
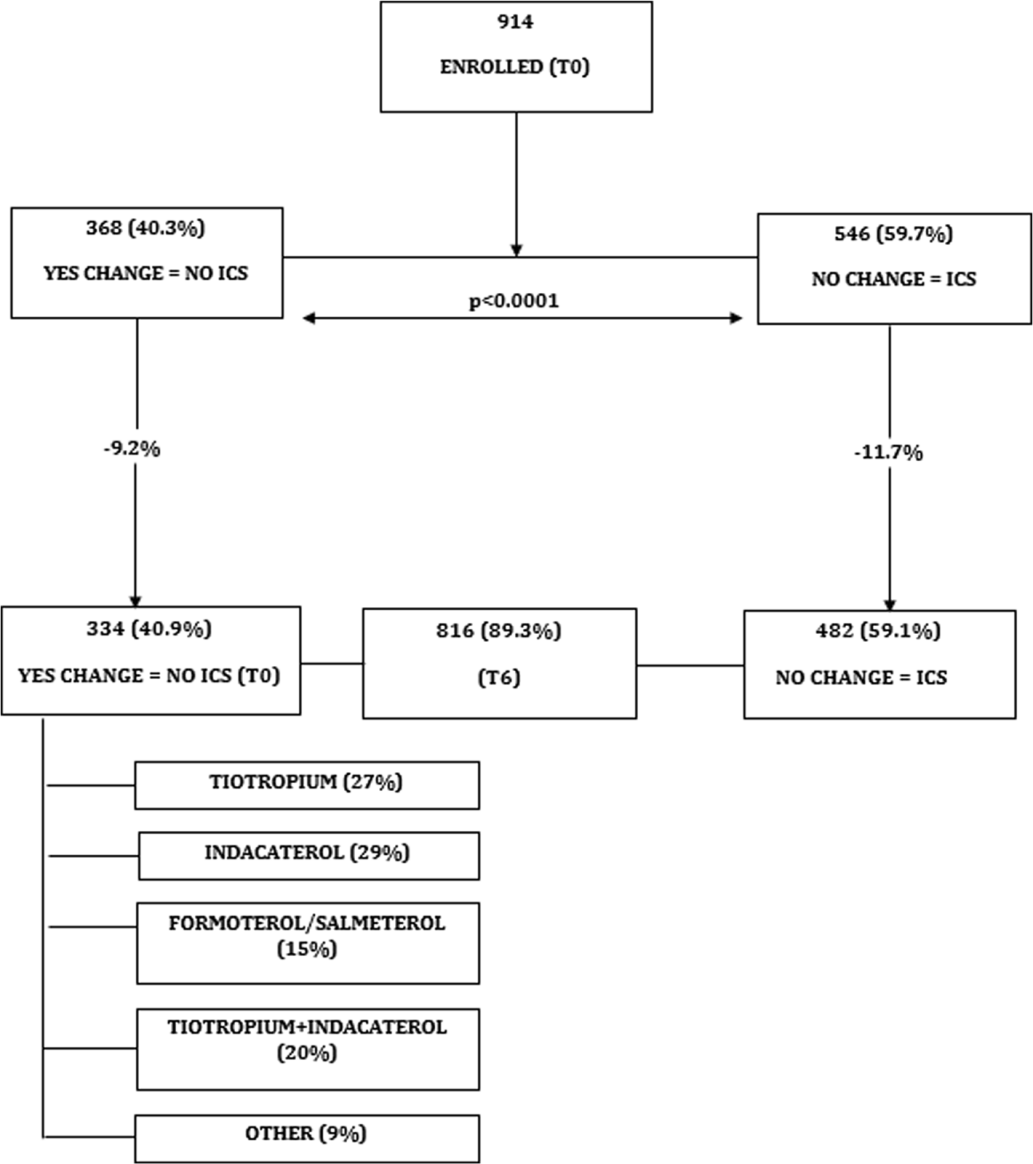
Study profile.

OPTIMO (Real-Life study On the aPpropriaTeness of treatment In MOderate COPD patients) was a multicenter, prospective, real-life study. Each pulmonary unit nominated a pulmonologist to be in charge of the study, who then attended a one-day start-up meeting before the actual study began to discuss the protocol, documents and guidelines. Particular focus was directed on ACP-ACCP-ATS-ERS [2], GOLD 2007 [8], GOLD 2011 [4], NICE [9] as well as the Italian National Document on the Integrated Management of COPD [22]. From July 2011 to April 2012, a total of 914 COPD patients were recruited in accordance with the inclusion and exclusion criteria. This was not to be a randomized clinical trial, but a prospective, real-life study and its design and flow chart are illustrated in Figure 1. Therefore, after having recruited the patients at the T0 visit, their physicians were free to prescribe whatever maintenance therapy they deemed fit. This information was then sent to the central data analysis office in AIPO (Associazione Italiana Pneumologi Ospedalieri) based in Milan, Italy. An intermediate optional visit three months on from enrollment could then be arranged, according to the clinical practice of the centers. However, the patients were encouraged to contact their pneumological center for any particular need or query during the study. A final visit was scheduled for all patients six months on from enrollment (T6).

### Patients

Patients were recruited in 43 centers in Italy (pulmonary units in both general and university hospitals), by their own treating physicians, all of whom have had first-hand experience of cooperating in real life studies [5]. Inclusion criteria of entry to the study meant that patients could be either gender, aged >40 years with an established history of COPD and with spirometric evidence of airflow obstruction. This meant a post-bronchodilator FEV_1_/VC ratio <88% and <89% predicted for men and women, respectively [23,24], and FEV_1_>50% predicted having suffered less than 2 exacerbations in the year prior to the study. The diagnosis of COPD was reported in the patient’s case report form (CRF) by the caring pulmonologist and based on patient’s clinical history and spirometric evidence of post-bronchodilator airflow obstruction. The latter was defined according to the ERS-COPD document [23] and the ERS/ATS criteria [24]. However, as expected on the basis of previous analysis [25] all our patients had a FEV_1_/VC ratio <0.70, basically in line with the GOLD recommendation [4,8], and very similar to the spirometric criteria in the COSMIC trial [20]. Patients were excluded if they had either a clinical diagnosis or a history of asthma, if they were on long-term oxygen therapy or if any COPD exacerbation or respiratory infection had occurred in the month before the recruitment visit (T0), if they had had serious uncontrolled psychological disease, myocardial infarction, acute heart failure, or angina pectoris in the six months preceding the visit. Patients with any kind of history of alcohol or drug abuse were also excluded. All of the above-listed characteristics had been ascertained at the visit preceding recruitment. Patients who met all the above criteria and who, according to their clinical record, had been on regular treatment with ICS in the previous year, were recruited at the T0 visit. At the same time, their physicians filled in a pre-established CRF entering all the information required for the study.

### Measurements

Forced Expiratory Volume in one second (FEV_1_) was calculated from the forced expiratory maneuver following a slow Vital Capacity (VC) maneuver in accordance with international protocol [24], and compared to ERS predicted values [26].

A COPD exacerbation was defined according to the ECLIPSE study [27], i.e. a change in symptoms leading to a brief course of antibiotics or systemic corticosteroids or both, depending on what the treating physicians deemed fit, and which was reported on the patient’s individual record. As patients did not receive a diary card, which would be the normal procedure in real life practice, mild exacerbations, i.e. a change in medication decided by the patients without consultation with her/his center, even over the phone, could have been missed: unreported exacerbations. However, it has to be noted that patients were instructed and actively encouraged to contact their center if they noticed their symptoms getting any worse. If the patient needed to be hospitalized, either at the discretion of the caring physician, or due to an emergency visit, exacerbation was considered severe.

Symptoms were assessed by means of the CAT (COPD Assessment Test, [28]) at the time of enrollment (T0) and at the final visit after 6 months (T6).

### Statistics

Results are expressed as mean and standard deviation if variables are continuous, and as a percentage if variables are categorical. Multiple regression model was used to compare continuous variables (FEV_1_ and CAT) in the different groups, adjusting for the confounders. Negative binomial regression, for over-dispersed count data, was used to compare the number of exacerbations in the different groups, adjusting for the confounders.

A p-value <0.05 was to be considered statistically significant. Analyses were performed using STATA (StataCorp, College Station, TX, USA) version 12.0.

## Results

Nine-hundred and fourteen COPD patients, on maintenance therapy with LABA plus ICS in the preceding year, from 43 pneumological centers in Italy were enrolled. Five-hundred and ten patients (56%) were on treatment with fluticasone/salmeterol 500/50 mcg bid, 165 patients (18%) with budesonide/formoterol 400/12 mcg bid, 112 patients (12%) with beclometasone/formoterol 200/12mcg bid, and 127 patients (14%) were taking ICS and LABA from different inhalers. 341 patients (37%) did not report any exacerbation in the year preceding T0, whereas 573 patients (63%) reported one exacerbation, in line with the inclusion criteria. At the time of enrollment, 109 patients (12%) had reported one hospitalization in the preceding year, although this was not due to respiratory-related causes in 25 of these. Therefore, 84 patients had one hospital admission related to their respiratory condition. The treating physicians decided to continue the treatment with ICS in 546 patients (59.7%) whereas ICS were withdrawn in 368 patients (40.3%).

Eight-hundred and sixteen patients (89.3%) concluded the study 6 months later, as illustrated in Figure 1. 482/816 patients (59%) continued their treatment with LABA/ICS with the same maintenance regimen as in the previous year (ICS group), whereas in 334/816 (41%) patients ICS were withdrawn and the treatment switched mainly to long acting bronchodilators (no-ICS group). Table 1 shows the characteristics of the 914 patients who started the study and the 816 patients who concluded the study, for the two groups of no-ICS (change in treatment) and ICS (no change in treatment) patients. The drop-out was 10.7% on the whole population, and 9.2% and 11.7% in the “no-ICS” and “ICS” groups, respectively. In the ICS group there is a higher prevalence of ex-smokers, and a slightly greater VC% predicted. In the starting population, more patients in the no-ICS group (32%) did not report comorbidities compared to the ICS group (24%). However, this difference was not observed in the groups of patients who concluded the study. Nevertheless, when we separated the kind of comorbidities, as shown in Table 2, the cardiovascular comorbidities (cardiac failure, ischemic hearth disorders, systemic arterial hypertension) were more present in the ICS group compared to the no-ICS group. No other difference was observed. In particular, the two groups were comparable at baseline for the variables analyzed in the study, namely CAT, FEV_1_, and exacerbations.

**Table 1:** Characteristics of the population who were enrolled at baseline and who concluded the study

**Total population**		**Enrolled (n = 914)**			**Concluding the study (n = 816)**		
**Groups**	**NO ICS (n = 368)**	**ICS (n = 546)**	**p-value**	**NO ICS (n = 334)**	**ICS (n = 482)**	**p-value**	
**Gender (%F)**	30%	27%	0.4102	30%	27%	0.2817
**Age (Yrs)**	72.0 (9.5)	72.8 (9.1)	0.1750	72.1 (9.2)	73.0 (8.9)	0.1936
**BMI**	27.6 (5.1)	28.3 (5.1)	0.0600	27.8 (5.1)	28.4 (5.1)	0.1458
**Smoking history° (%Y)**	82%	91%	0.0007	83%	91%	0.0019
**Ex-smokers**	60%	69%	0.0047	60%	68%	0.0187
**Smokers**	22%	22%	1.0000	23%	23%	1.0000
**Years since diagnosis**	6.8 (6.7)	6.6 (6.0)	0.5401	6.8 (6.6)	6.6 (6.0)	0.6794
**VC (%pred)**	91.5 (15.9)	95.2 (19.5)	0.0054	91.4 (15.7)	94.7 (18.6)	0.0124
**FEV**_**1 **_**(%pred)**	71.7 (10.4)	70.8 (11.3)	0.2608	71.6 (10.5)	71.1 (11.4)	0.5293
**FEV**_**1**_**/VC**	0.60 (0.08)	0.59 (0.08)	0.2679	0.60 (0.08)	0.59 (0.08)	0.1865
**CAT**	15.9 (8.5)	14.8 (7.8)	0.0717	16.0 (8.6)	14.8 (7.8)	0.0528
**Exacerbations (0/1)**	135/233	206/340	0.802	127/207	186/296	0.928
**Comorbidities: 0**	116 (32%)	130 (24%)	0.012	102 (30%)	109 (23%)	0.285
**Comorbidities: 1**	110 (30%)	174 (32%)	0.575	101 (30%)	153 (32%)	0.800
**Comorbidities: ≥2**	142 (38%)	242 (44%)	0.088	131 (40%)	220 (45%)	0.390

Data are presented as N, unless otherwise stated. Values are expressed as mean (SD).

BMI: Body Mass Index; VC: Vital Capacity; FEV_1_: Forced Expiratory Volume in 1 second; CAT: COPD Assessment Test.

**°**Y = includes currents smokers and ex-smokers.

**Table 2:** Comorbidities in the two groups who concluded the study (n = 816)

	**NO ICS (n = 334)**	**ICS (n = 334)**	**p-value**
**Cardiovascular**	52%	64%	0.001
**Obesity**	17%	19%	0.523
**Gastric reflux**	13%	14%	0.918
**Diabetes**	13%	12%	0.787

**Figure 2:**
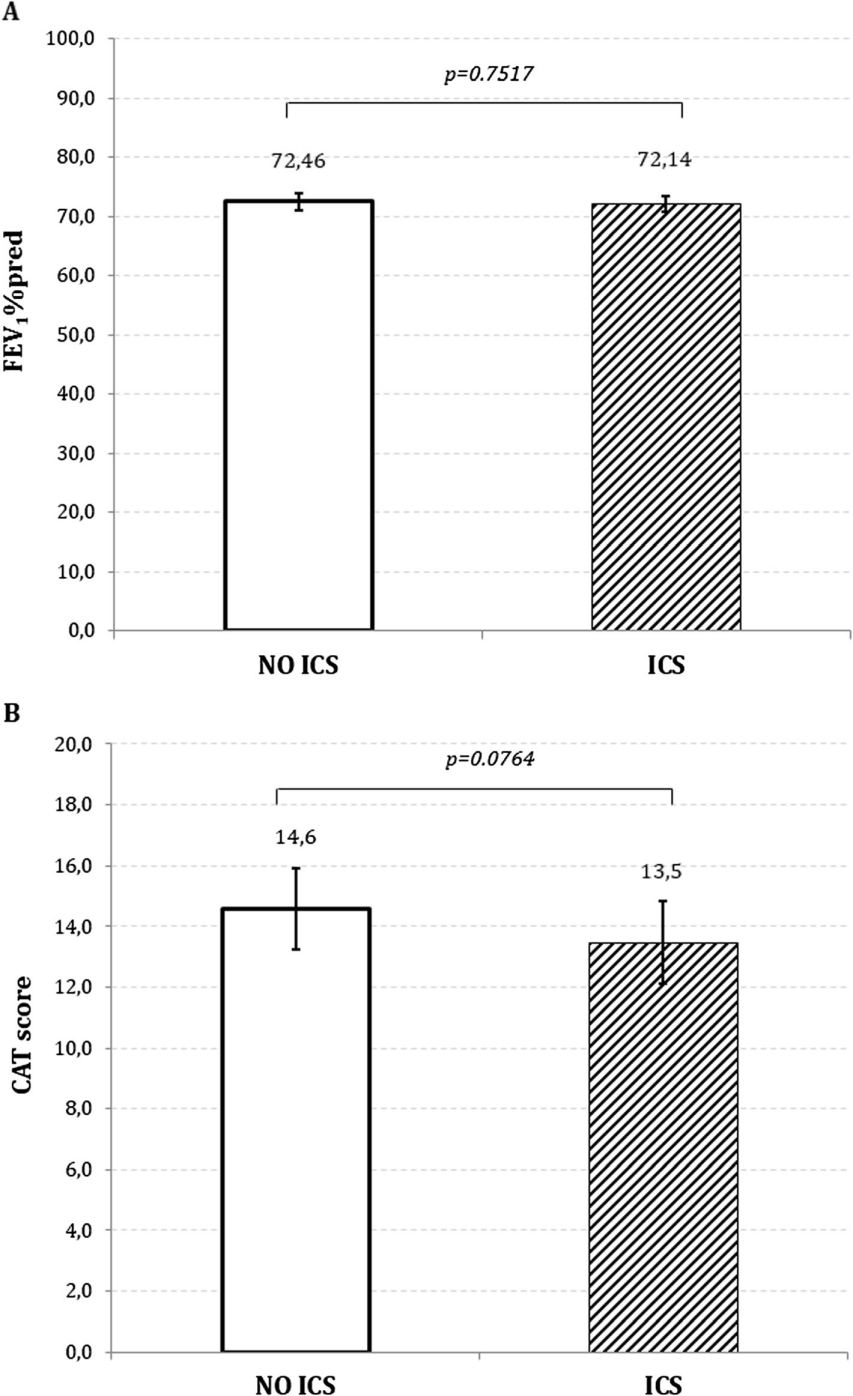
**Mean (SE) of FEV**_**1**_**% predicted (A) and of CAT score (B) at T6 in the two groups of patients who were switched to long acting bronchodilators or continued their treatment with ICS.**

**Figure 3:**
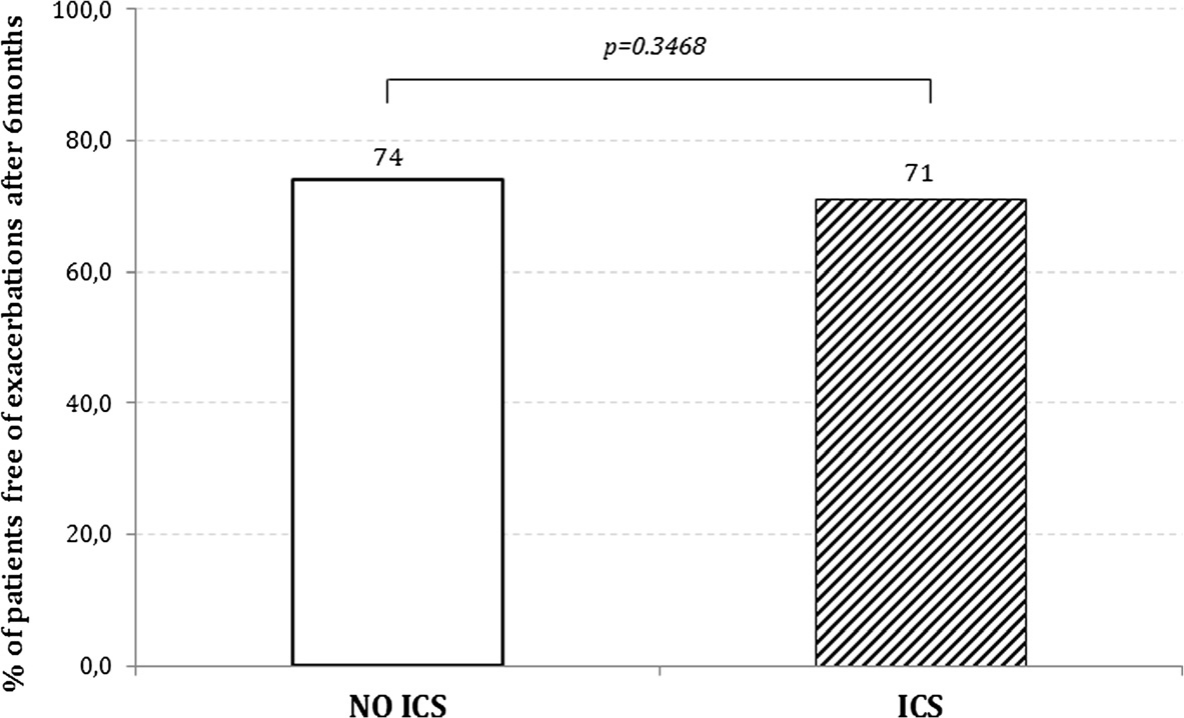
Percentage of patients without exacerbations at the end of the study (T6).

**Figure 4:**
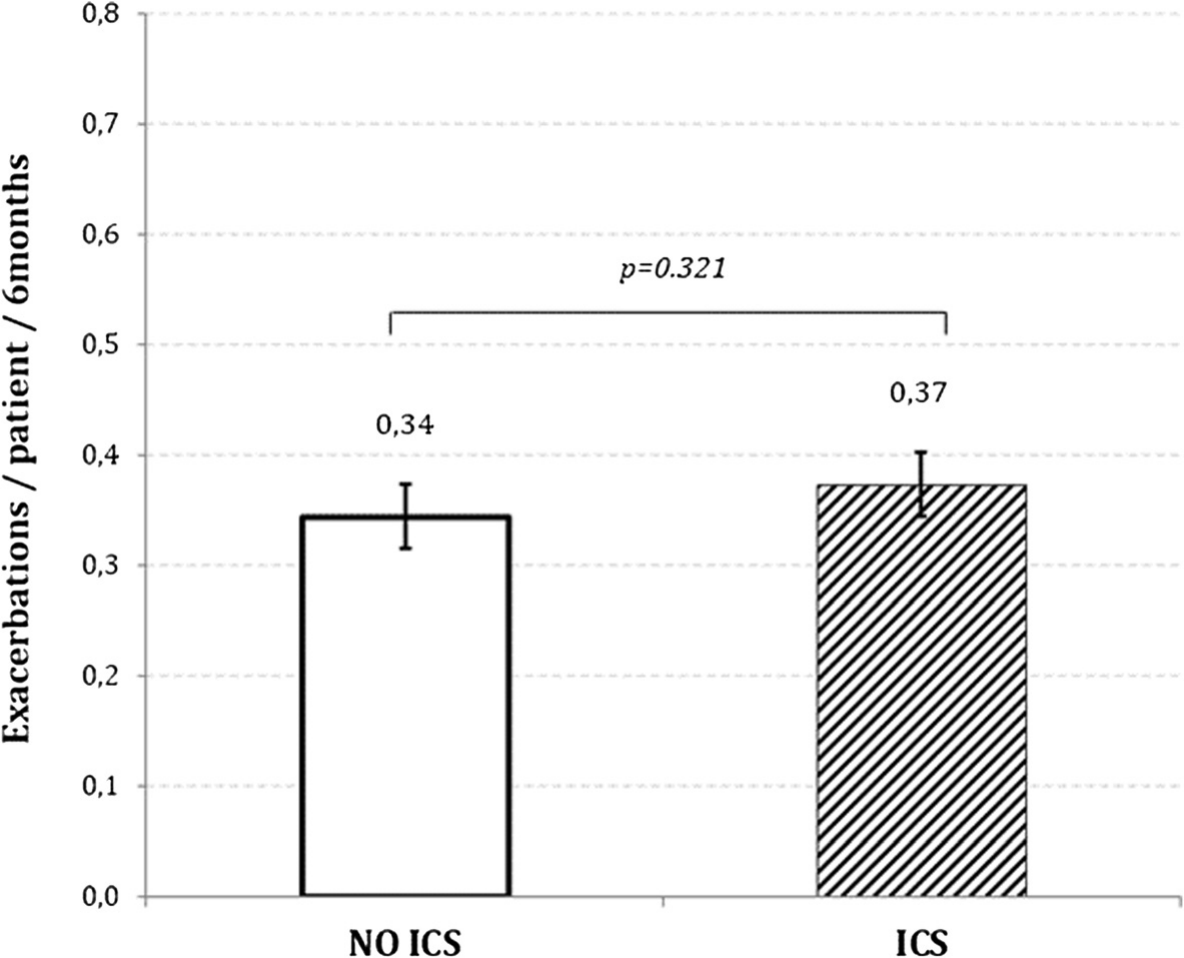
Mean (SE) exacerbation rate expressed as exacerbation/patient/6 months at the end of the study (T6) in the two groups of patients who were switched to long acting bronchodilators (NO ICS) OR continued the treatment with ICS.

CAT and FEV_1_ did not change from T0 to T6 in either group and no difference was found between the groups at T6 (Figure 2A and B). During the six months of the study, 229/816 patients (28%) reported at least one exacerbation. In this group, 185 patients reported one exacerbation while 44 patients reported >1 exacerbation: 35 patients, 6 patients, and 3 patients reported 2 exacerbations, 3 exacerbations, and 4 exacerbations, respectively. Therefore, the total number of exacerbations in the six months of the study amounted to 285 exacerbations. 141/482 patients (29%) and 88/334 patients (26%) exacerbated in the group treated with ICS and without ICS, respectively (Figure 3). In all, 173 exacerbations were reported in the ICS group and 112 in the non-ICS group, i.e. 0.37 and 0.34 exacerbation/patient/6 months. As illustrated in Figure 4, there is no significant difference (p = 0.321). Since the exacerbation rate distribution had a high variability (standard deviation greater than the mean), we analyzed the data with negative binomial regression model for over dispersed count data. The RR of the two groups (ICS vs NO ICS) is not significantly different (p = 0.321) after adjusting for confounding factors (age, smoke habits, FEV_1 _%pred (T0), BMI and gender) as illustrate in Table 3. The goodness of the model, compared to the Poisson regression model, is confirmed by the fact that the alpha parameter is significantly different from zero.

**Table 3:** Negative binomial regression model for over dispersed count data (numbers of exacerbations)

**Exacerbations (T6)**	**RR (95% CI)**	**Std. err.**	**p-value**
ICS*	0.8777 (0.679-1.135)	0.115	0.321
**Age**	1.004 (0.999-1.018)	0.007	0.594
**Smoke habits**	0.834 (0.576-1.207)	0.157	0.335
**FEV**_**1**_**%pred (T0)**	1.008 (0.997-1.020)	0.006	0.170
**CAT (T0)**	1.035 (1.020-1.052)	0.008	0.000
**BMI**	0.980 (0.956-1.005)	0.013	0.122
**Gender**	1.043 (0.786-1.385)	0.151	0.768
**Constant**	0.174 (0.343-0.881)	0.144	0.058
Alpha**	0.239 (0.065-0.878)	0.159	0.043

(pseudo R^2^ = 0.0199; p-value = 0.0012).

*reference group “NO ICS”.

**parameter for over dispersed count data (likelihood-ratio test of alpha = 0).

Forty-two patients, who did not exacerbate in the previous year reported ≥1 exacerbations in the 6 months of observation: 18/334 (5%) and 24/482 (5.3%) in the no-ICS and ICS group respectively. On the other hand, 34% and 39% of patients in the ICS and no-ICS group respectively, went from 1 to 0 exacerbations. No significant difference. Twenty-seven patients were hospitalized, twenty of which due to respiratory-related causes: 15 patients in the ICS group (3.1% of 482), and 5 in the no-ICS group (1.5% of 334).

**Figure 5:**
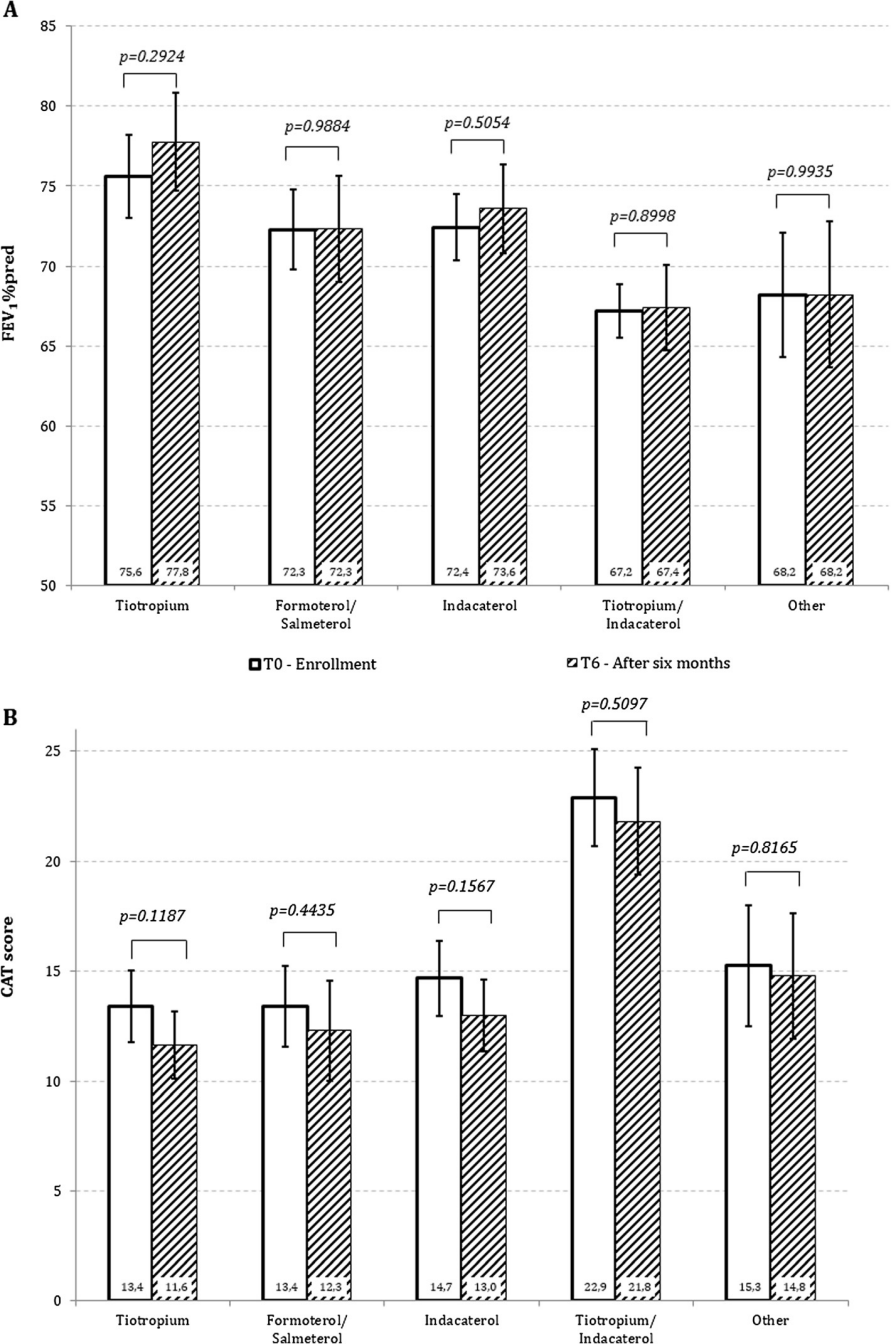
**Mean (SE) of FEV**_**1 **_**% predicted (A) and of CAT score (B) at T0 and T6 in the different groups of treatments (NO ICS).**

As shown in Figure 1, among the 334 patients in whom the initial LABA/ICS treatment was changed, monotherapy with tiotropium 18mcg/die or LABA was instituted for 89 patients (27%) and 147 patients (44%), respectively. In this latter group, 98 (29%) patients received indacaterol 150mcg/die, and 49 patients (15%) were treated with either formoterol 12mcg/bid or salmeterol 50mcg/bid. In 67 patients (20%) the association of tiotropium 18mcg with indacaterol 150mcg was prescribed for regular treatment. In 31 patients (9%) the treating physicians prescribed either short-acting bronchodilators, in general salbutamol, and/or theophylline. Therefore, in the majority of patients, the therapy was “downgraded” from the LABA-ICS combination to a bronchodilator monotherapy. In a minority of patients the therapy was switched to the association of two long acting bronchodilators. Table 4 shows the baseline characteristics of the patients in the different groups of changed treatment. The distribution of patients with exacerbations at T0 among the different groups of treatment, after ICS withdrawal is also shown in Table 4. The prevalence of exacerbations was 66% and 54% in the “downgraded” (monotherapy) and in the tiotropium + indacaterol groups, respectively. After the 6 months of observation, the prevalence of exacerbations at T6 was as follows: tiotropium 27/89 (30%), LABA 32/147 (21%), indacaterol 22/98 (22%), and other 13/31 (42%), with a total of 35% (94/267) in the “downgraded” group compared to a 24% (16/67) in the tiotropium + indacaterol group. The number of patients in each group is small and too different in magnitude for any additional statistical comparison, which however was not in the scope of this study. Furthermore, the data in Table 4 refer to one year preceding the study, while the data at the end of the study refer to 6 months only. It is noticeable that the patients switched to the tiotropium plus indacaterol combination had, on average, the higher CAT and lower FEV_1_ %predicted compared to the other groups. Figure 5 (A and B) shows the data of mean FEV_1_ and CAT at T0 and T6 for the groups. No significant difference.

**Table 4:** Baseline characteristics of patients in the different groups of treatments (NO ICS)

	**NO ICS (n = 334)**				
	**Tiotropium (n = 89)**	**Formoterol/Salmeterol (n = 49)**	**Indacaterol (n = 98)**	**Tiotropium+Indacaterol (n = 67)**	**Other (n = 31)**
**Gender (%F)**	28%	25%	28%	33%	36%
**Age (Yrs)**	72.0 (9.6)	71.5 (8.1)	71.4 (10.0)	73.4 (9.7)	71.7 (9.4)
**BMI**	27.4 (4.8)	27.2 (4.8)	27.5 (4.9)	28.3 (5.6)	28.0 (5.3)
**Smoking history° (%Y)**	79%	78%	84%	86%	83%
**Years since diagnosis**	5.4 (6.6)	8.7 (8.6)	7.3 (5.8)	6.4 (6.3)	7.2 (7.0)
**VC (%pred)**	96.1 (16.7)	90.5 (15.9)	91.2 (14.8)	88.6 (14.9)	89.4 (17.0)
**FEV**_**1 **_**(%pred)**	75.2 (15.6)	72.0 (8.2)	72.8 (10.2)	67.5 (6.9)	68.1 (12.1)
**FEV**_**1**_**/VC**	0.61 (0.10)	0.60 (0.08)	0.60 (0.08)	0.59 (0.08)	0.59 (0.04)
**CAT**	13.8 (7.2)	13.3 (5.8)	14.7 (8.0)	21.9 (9.2)	15.0 (8.6)
**Exacerbations (0/1)**	35/54	13/36	33/65	31/36	10/21
**Comorbidities: 0**	31 (35%)	20 (41%)	32 (33%)	17 (26%)	6 (19%)
**Comorbidities: 1**	23 (26%)	16 (33%)	27 (27%)	24 (36%)	10 (31%)
**Comorbidities: ≥2**	35 (39%)	13 (26%)	39 (40%)	26 (38%)	15 (50%)

Data are presented as N, unless otherwise stated. Values are expressed as mean (SD).

BMI: Body Mass Index; VC: Vital Capacity; FEV_1_: Forced Expiratory Volume in 1 second; CAT: COPD Assessment Test.

**°**Y = includes current smokers and ex-smokers.

In the year preceding the enrollment, 17 patients reported an episode of pneumonia. During the six months of observation, 3 new cases of pneumonia were reported: two in the group without ICS and one in the group continuing ICS treatment. We did not observe any death in the six months of the study.

## Discussion

This real-life, prospective study shows that withdrawal of ICS in symptomatic COPD patients with moderate airflow limitation, i.e. FEV_1_>50% predicted, and no history of frequent exacerbations, i.e. having suffered less than 2 exacerbations in the year prior to the study, was not associated with any deterioration in symptoms, lung function, and exacerbation rate during six months of observation. The data were analyzed having adjusted for all the confounding variables at baseline. Although a minority (9%) of patients were switched to short-acting bronchodilators and/or theophylline, the substantial majority of the patients (91%) were switched to long-acting bronchodilators, either in monotherapy (71%) or in association (20%). To the best of our knowledge, this is the first study to show that withdrawal of ICS in COPD patients for whom ICS are not recommended by international documents and guidelines can be safe provided that the patients remain on regular treatment, for the most part with long-acting bronchodilators. The results of this real-life study support the recommendation of international documents [4] and guidelines [9] that regular treatment with ICS is not needed in COPD patients who are at low risk of exacerbations.

It is interesting to note that, though the mean FEV_1_ was greater than 50% predicted in all patients in this study, the average CAT value was greater than 10 points. In other words, the COPD patients in this study had moderate-to-mild airflow obstruction, but they did report symptoms. They could be classified as patients B in the new GOLD categories [4]. However, this is of no great surprise as the elegant work by Ofir and colleagues [29] showed that COPD patients with mild airflow obstruction, GOLD stage 1 [8], had a lower exercise tolerance than control subjects and that they interrupted their exercise mostly for breathlessness, whereas control subjects stopped mainly for leg fatigue.

Exactly what benefits COPD patients can hope for from regular treatment with ICS remains controversial [21] and a discussion on this issue goes well beyond the scope of this study which aimed to investigate the consequence of ICS withdrawal in “low-risk” COPD patients. We acknowledge that the lack of randomization is a major limitation of this study and that we cannot draw a definitive conclusion which requires a randomized controlled trial. However, Table 1 shows that the only differences between the two groups of patients who concluded the study are the lower prevalence of ex-smokers and the slightly lower VC% predicted, although it was in the normal range, in the no-ICS population. However, Table 3 shows that smoking habits did not have any significant effect on the occurrence of exacerbations. Furthermore, although the prevalence of patients with comorbidities did not differ between the two groups of patients who concluded the study, the presence of cardiovascular comorbidities was greater in the ICS group. It is possible that the presence of cardiovascular comorbidities could have influenced the decision of the caring pulmonologists at T0 not to discontinue the ICS treatment. However, for all the other variables such as age, gender, BMI, years since diagnosis, and in particular for the variables specifically examined in this study, namely CAT, FEV_1_, and exacerbations, there was no significant difference either at the beginning or at the end of the study between the no-ICS and ICS groups of patients. Furthermore, the population of COPD patients enrolled in this study is rather numerous compared to the other, randomized, trials on the same topic [17-20].

It is of note that, in this real-life study, some interesting information comes from the fact that the treating pulmonologists were free to choose the maintenance treatment for their patients. In fact, the majority decided to continue the regular therapy with ICS, despite the wide discussion on the explicit recommendations from international [2,4,8,9] and national [10,22] documents. It cannot be said therefore that doctors, and even specialists, fail to follow the guidelines due to a lack of available information. There are other explanations which need to be investigated [21]. It seems not to be a problem specific to a single country such as Italy: in the multi-centric and multi-country ECLIPSE [27] and UPLIFT studies [30], about 60% of patients in GOLD stage 2 were treated with ICS.

The effect of discontinuing ICS in patients with COPD was investigated in a few studies [31]. In the COPE study [18], 244 COPD patients who were on regular mono-therapy with fluticasone 500 mcg/bid were randomized either to continue fluticasone (123 patients) or to receive placebo (121 patients) for 6 months. In the placebo arm there was a higher recurrence-risk of exacerbations and a significant deterioration in health-related quality of life. The WISP study [19] was conducted in the primary care setting. 260 COPD patients were taken off their usual ICS therapy and were administered 500mcg fluticasone bid (128 patients) or placebo (132 patients) for one year. The risk of exacerbation increased in patients withdrawn from ICS and was associated with a deterioration in symptoms. Similar results were found in a smaller 6-week study: ventilatory function and dyspnea worsened in severe COPD patients when ICS were withdrawn [17]. However, those studies compared ICS to a placebo arm.

In the COSMIC study, Wouters and colleagues [20] enrolled almost 500 COPD patients after three months of treatment with the fluticasone/salmeterol 500/50mcg bid combination and monitored them for 1 year after randomization to a continuation arm (189 patients) or to a salmeterol 50mcg bid monotherapy arm (184 patients). They found that the switch from the fluticasone/salmeterol combination to salmeterol alone resulted in persistent deterioration of lung function and dyspnea and in an increase in mild exacerbations, while there was no significant difference for moderate-to-severe exacerbations. To our knowledge, the COSMIC is the only study in which patients remained on regular treatment with a long-acting bronchodilator after withdrawal of ICS. However, the patients in the COSMIC study had 2 or more exacerbations in the preceding year and mean FEV_1_ was less that 50% predicted at both enrollment and randomization. By contrast, the patients in our study had a FEV_1_ >50% predicted, the FEV_1_ amounting on average to 71.2% predicted in the whole population, and less than 2 exacerbations in the preceding year was a criterion for enrollment. Therefore, whereas the patients in the COSMIC study met the basic criteria for the ICS/LABA combination treatment, the patients in our study can be defined as “low risk” patients for exacerbations according to the classic (GOLD II) [8] as well as to the most recent GOLD classification (GOLD B) [4]. In addition to the difference in patient populations, i.e. “high risk” in the COSMIC versus “low risk” in this study, there was also a difference in protocol design. Our study is a prospective, real-life survey in the secondary care environment, not a randomized trial. Furthermore, the COSMIC was a one-year trial [20], whereas our study lasted 6 months. However, the deterioration in lung function and symptoms in the salmeterol monotherapy group in the COSMIC was already clear well before 6 months. In this regard, it should be noted that the COPE was also a 6-months study [18]. Therefore, we believe that the results of our study, which showed no deterioration in the no-ICS group, may be considered valid. In addition it has to be considered that the recruiting period, in our study, went from July 2011 to April 2012, hence including both a summer and a winter season. In the COSMIC study, no difference was observed between arms for the moderate-to-severe exacerbations, whereas an increase was reported in the rate of mild exacerbations [20]. Therefore the result on moderate and severe exacerbations is similar in the two studies. We did not examined specifically the issue of mild exacerbations. The patients did not have a diary card. However, all the changes in symptoms that patients reported to their caring pulmonologists at the referring center were recorded. No such increase was observed in our study. Whether some patients had the “unreported exacerbations” remains unknown in this as well as in many other studies.

Unlike the COSMIC trial, clinicians in our study were free to choose which regular treatment to prescribe to their patients after withdrawing ICS. In the vast majority of cases, doctors prescribed regular treatment with long acting bronchodilators (91%) either in mono-therapy or in combination. Therefore, in the majority of patients, the change was a downgrade to a long acting bronchodilator mono therapy (236/334, 71%) whereas in 67 patients (20%) the LABA/ICS combination was switched to a tiotropium + indacaterol association. Table 4 shows that the patients in the latter group had, on average, a lower FEV_1_ % predicted, a higher CAT score, but not a greater occurrence of exacerbations. The analysis of these data goes beyond the possibility of the present study. In fact, we addressed the issue of ICS withdrawal not the subsequent therapeutic choice. However, the data on Table 4 might suggest that doctors upgrade the bronchodilating therapy in patients with a worse FEV_1_ and more symptoms. A recent study has shown that the combination of indacaterol and tiotropium provides greater therapeutic benefits than tiotropium alone [32].

In the ILLUMINATE study [33] the once-daily QVA149 (indacaterol 110 μg and glycopyrronium 50 μg) was compared with salmeterol-fluticasone 50/500 μg bid fixed dose combination. For the QVA149 arm, the ICS had been withdrawn. No deterioration of symptoms, lung function, and exacerbations was observed in comparison with the ICS arm. The patients of the ILLUMINATE study were at low risk as the patients of our study. We believe that those data from ILLUMINATE support our conclusion.

In our study, hospital admissions were numerically more frequent in the ICS than in the non-ICS group: 15 (3.1%) vs 5 (1.8%). This might be due to the greater prevalence of cardiovascular comorbidities in the ICS group (Table 2). Only a few cases of pneumonia were reported during the six months of observation. However, it is known that pneumonia may represent a risk for prolonged treatment with ICS in COPD patients [13-16].

## Conclusions

This real-life, prospective study suggests that the withdrawal of ICS in patients at low risk of exacerbation, namely patients with moderate airflow limitation (FEV_1_ >50% predicted) and having suffered less than two exacerbations in the year preceding the study, can be safe provided that patients are left on maintenance treatment with long-acting bronchodilators. Although this is not a randomized controlled trial, the data support this conclusion and can stimulate a controlled clinical trial on this important issue in respiratory medicine.

## Abbreviations

ACCP: American college of chest physicians; ACP: American college of physicians; AIPO: Associazione Italiana pneumologi ospedalieri; ATS: American thoracic society; BMI: Body mass index; CAT: COPD assessment test; COPD: Chronic obstructive pulmonary disease; CRF: Case report form; ERS: European respiratory society; FDC: Fixed dose combination; FEV_1_: Forced expiratory volume in one second; GOLD: Global initiative for chronic obstructive lung disease; ICS: Inhaled corticosteroids; LABA: Long-acting beta-adrenergic agonists; LAMA: Long-acting antimuscarinic agents; NICE: National institute for health and care excellence; VC: Vital capacity.

## Competing interests

AR reports personal fees and non-financial support from Astra Zeneca and Boehringer Ingelheim, grants and personal fees from Chiesi, personal fees from Glaxo Smith Kline, grants, personal fees and non-financial support from Novartis, outside the submitted work. MG and AC declare that they have no competing interests.

## Authors’ contributions

AR and AC realized the study design and supervised the data collection. AR, AC and MG contributed to the data analysis, writing of the manuscript, data interpretation, discussion and answers to Reviewers. All of the authors read and approved the final manuscript.
